# Inulin Supplementation Reduces Systolic Blood Pressure in Women with Breast Cancer Undergoing Neoadjuvant Chemotherapy

**DOI:** 10.1155/2019/5707150

**Published:** 2019-07-01

**Authors:** Yizel Becerril-Alarcón, Saúl Campos-Gómez, Juan J. Valdez-Andrade, Karen A. Campos-Gómez, Diana Y. Reyes-Barretero, Alejandra D. Benítez-Arciniega, Roxana Valdés-Ramos, Alexandra E. Soto-Piña

**Affiliations:** ^1^Facultad de Medicina, Universidad Autónoma del Estado de México, Mexico; ^2^Departamento de Oncología Médica, Centro Oncológico Estatal ISSEMYM, Mexico

## Abstract

**Introduction:**

Breast cancer is the most frequently diagnosed malignancy in women, and comorbidities like hypertension and obesity diminish their quality of life and negatively affect their response to chemotherapy. Furthermore, inulin supplementation is associated with the reduction of cardiovascular diseases (CVD) risk.

**Objective:**

To determine whether inulin supplementation prevents the elevation of blood pressure in women with breast cancer undergoing neoadjuvant therapy with cyclophosphamide and doxorubicin.

**Methods:**

This was a randomized, double-blind placebo controlled trial which included women with early-stage breast cancer undergoing neoadjuvant therapy (n=38). Patients were randomly assigned to participate in two different groups to receive either 15 g of inulin or 15 g of placebo (maltodextrin) for 21 days. Body composition and blood pressure were evaluated before and after the supplementation period.

**Results:**

Women in the inulin group showed a lower systolic blood pressure (SBP) after the supplementation (-4.21 mmHg,* p*<0.001). However, SBP increased in the placebo supplemented group. Diastolic blood pressure (DBP) nonsignificantly decreased in the inulin group. Inulin supplementation also increased BMI (*p*<0.001) but reduced BFP (*p*=0.288). Furthermore, confounding variables, such as BMI, baseline fasting glucose, age, menopause status, vomiting, constipation, and chronic medication did not have a statistical influence over the inulin effect on SBP.

**Conclusion:**

Inulin supplementation reduces SBP and prevents increases in DBP in women with breast cancer. This could be an innovative nutraceutical approach to prevent hypertension present in women with this type of cancer at an early stage and may improve the quality of life of the patients and their prognostic development through chemotherapy.

**Trial Registration Number:**

This trial is registered with ACTRN12616001532493.

## 1. Introduction

Women with breast cancer frequently have comorbidities, such as hypertension and other conditions related to body composition, which diminish their quality of life and negatively affect the response to treatment. Hypertension is the comorbidity associated with breast cancer with the highest prevalence (21.8%), which is followed by chronic pulmonary obstruction (19.9%) and diabetes mellitus (16.7%) [1]. The presence of hypertension could be related to age and treatment [2]. Patients with breast cancer under adjuvant therapies such as radiotherapy or chemotherapy may have an increased risk of cardiac toxicity and cardiovascular disease (CVD), which has become an important cause of death among women with breast cancer, representing 10% of mortality [3]. The use of some chemotherapeutic agents and doses has been associated with CVD; according to the National Surgical Breast and Bowel Project B-31, 17% of the patients treated with doxorubicin and cyclophosphamide develop asymptomatic heart diseases, associated with accumulated doses lower than 500 mg/m^2^ [4, 5]. In addition, gastrointestinal adverse events, such as vomit and diarrhea, could appear due to chemotherapy; these can lead to alterations in blood volume and systemic vascular resistance and consequently affect blood pressure [6]. The nutritional status of patients is important in the response to treatment as well. Weight gain is common in patients with breast cancer, especially in women receiving adjuvant chemotherapy; the magnitude of this effect is variable and could be related to the types of chemotherapeutic agents, doses, duration of the treatment, and menopausal state [7].

The origin of hypertension in patients with cancer is not clear; it could appear before cancer diagnosis or after that. For instance, women who used antihypertensive medications have an increased risk of breast cancer [8]. Also, chemical agents used in neoadjuvant therapy such as doxorubicin and cyclophosphamide could generate a cardiotoxic effect, which may be a risk factor for developing high blood pressure, during the treatment. However, cardiac damage and symptoms may appear up to five months after the administration of the last dose [9]. One of the approaches to treat or prevent the cardiotoxicity induced by doxorubicin and cyclophosphamide is the reduction of reactive oxygen species using drugs like dexrazoxane [10] or phytochemicals and soluble fiber that have shown similar effects [11].

Diet components can play a regulatory role in the development of hypertension as well. Particularly, some vegetables contain fiber and prebiotics that modulate blood pressure, preventing the appearance of hypertension [12]. For instance, inulin is a prebiotic that enhances intestinal health and immune system function especially, by selectively increasing the growth of bifidobacteria [13] and reducing toxic metabolites [14]. Moreover, the benefits of inulin in metabolic and cardiovascular function are associated with effects on gut dysbiosis, secretion of incretins, dyslipidemia, and obesity control [15–18]. Some of these, like the improvement of insulin sensitivity, regulation of incretin secretion, and reduction of hepatic triacylglycerols, are mediated by Short Chain Fatty Acids (SCFA), which are the products of inulin fermentation in the gut [19–21]. Interestingly the administration of SCFA such as propionate reduces cardiac hypertrophy and vascular dysfunction in hypertensive mice [22]. In women with diabetes, inulin supplementation reduces cardiometabolic risk and enhances antioxidant capacity [23].

Prebiotics provide a variety of health benefits like the ability to increase concentrations of vitamin B and plasma glutamine, which could improve immune function [24]. In particular, inulin-type fructans reduce hypercholesterolemia [25] and revert endothelial dysfunction, an effect associated with the influence of microbiota on host intestinal function and cardiometabolic health [26]. In light of this, inulin supplementation could be a potential aid in the treatment of hypertension because of its multiple health benefits at a local and systemic level. Therefore, the aim of this study was to determine whether inulin supplementation prevents the elevation of blood pressure in women with breast cancer undergoing neoadjuvant therapy with cyclophosphamide and doxorubicin.

## 2. Methods

A randomized, double-blind trial was conducted, involving women (n=38) diagnosed with breast cancer at early stage, beneficiaries of the State Oncology Center of the Social Security Institute of the State of Mexico and Municipalities (ISSEMYM). The inclusion criteria were women aged >18 years and planned to receive neoadjuvant chemotherapy with doxorubicin and cyclophosphamide for the first time. The participants received a standard chemotherapeutic treatment, with cyclophosphamide and doxorubicin (600 mg/m^2^/ 60mg/m^2^), by infusion, as part of the medical scheme established in the State Cancer Center. Patients were randomly assigned to participate in two different groups to receive 15 g of powdered agave inulin (Latin Foods, Mexico City) or 15 g of maltodextrin (Latin Foods, Mexico City) as a placebo. Both products were supplied in sealed packages and previously codified by trained personnel in the Molecular Biology Laboratory of the Medical Sciences Research Center (CICMED) in Toluca, State of Mexico. Once participants were assigned to one of the two groups, they were provided with 21 packages according to their respective group. After that, patients from both groups were asked to dissolve the contents of an envelope in 240 mL of drinking water, to be consumed during the breakfast. Patients initiated the ingestion of the supplement on the same day that the first cycle of chemotherapy started, and they kept taking the respective supplement for 21 days. The study was conducted in accordance with the Declaration of Helsinki and the Mexican Legislation on Scientific Research in Humans; also, the study was registered in the Australian New Zealand Clinical Trials Registry with number ACTRN12616001532493, on November 7, 2016. The protocol was approved by the Ethics Committees of the ISSEMYM State Oncology Center and the School of Medicine of the Autonomous University of the State of Mexico. Written informed consent from each participant was obtained once every patient accepted to be included in the study.

### 2.1. Body Composition and Blood Pressure

The baseline assessment of body composition was performed on the first appointment to receive the first chemotherapy cycle and the second assessment at the end of the 21 days of supplementation. The assessment of body composition was performed using electric bioimpedance with the equipment InBody 230 (InBody Co., Seoul, South Korea). This analysis included body weight, body mass index (BMI), body fat percentage (BFP), lean body mass (LBM), and skeletal muscle mass (SMM), and it was conducted three hours after eating or drinking. To perform the measurement, patients were barefoot and stood on a platform at a 90° position facing ahead, holding both handles of the apparatus until the impedance scan finished and the analysis results were printed. Moreover, blood pressure was measured before and after supplementation (at the end of the 21 days). The procedure to measure blood pressure was performed according to the Official Mexican Norm for prevention, detection, diagnosis, treatment, and control of systemic arterial hypertension (NOM-030-SSA2-2009). Once systolic blood pressure (SBP), diastolic blood pressure (DBP), arterial mean pressure (MAP), and hear rate (HR) were measured, the results were compared between both study groups, before and after the supplementation period. HR was computed as the number of beats in 15 seconds by four, and it was expressed as beats per minute (bpm). The classification of patients according to blood pressure recordings was performed using the criteria established by the American College of Cardiology and the American Association of Hypertension. BMI and BFP classification was conducted according to the criteria established by World Health Organization (WHO) and to Bray G, et al. [27].

### 2.2. Clinical and Dietary Monitoring

Diet information and clinical data of renal function (urea and creatinine concentrations) were extracted from medical records in the State Oncology Center; the data corresponded to results from laboratory tests that were performed prior the beginning of the chemotherapy cycles. Furthermore, it is important to point out that, before starting chemotherapy, adequate renal and hepatic functions were verified in all participants. Regarding that, creatinine clearance was calculated with the Cockcroft-Gault equation. Dietary data were collected by the nutrition department, through an institutional questionnaire of habitual diet; the consumption was reported in equivalent measures according to the Mexican System of Equivalent Foods [28]. In addition, patients at the time of diagnosis received nutritional counselling throughout the period of treatment. The evaluation of gastrointestinal adverse events (vomiting and constipation) was carried out using the EORTC-QLQ-C30 questionnaire, and it was performed each week during the 21-day supplementation, recording the adverse events that had appeared since the administration of the first chemotherapy until the day of the second evaluation.

### 2.3. Statistical Analysis

The normality of SBP, DPB, MAP, HR, BMI, and BFP was assessed by the Kolmogorov-Smirnov test. Chi-square test was used to compare frequencies of blood pressure and body composition categories before and after supplementation between placebo and inulin groups. Measures of central tendency were obtained, and the effect size for each variable by group was computed as follows: (mean before supplementation - mean after supplementation)/ standard deviation. To identify significant mean differences between placebo and inulin groups a Student t-test was performed. Also, Mantel-Haenszel test was used to rule out the effect of confounders factors on blood pressure. All tests were performed in the statistical software SPSS 21, considering a significance level of 0.05.

## 3. Results

**Figure 1:**
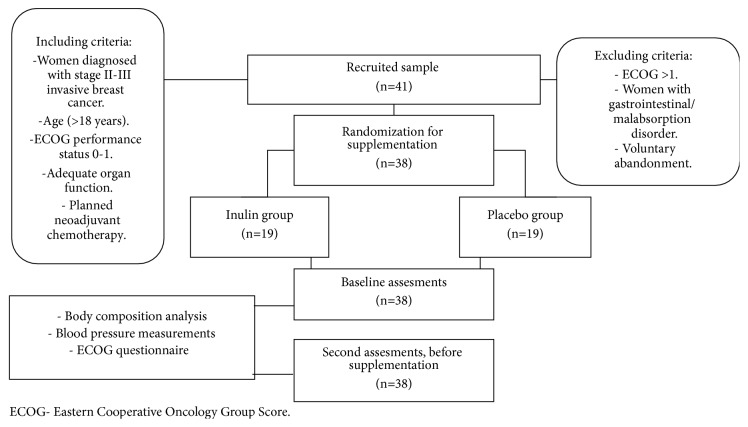
Study design and sample collection.

The original sample (Figure 1) included 41 women; however, only 38 women compelled with the inclusion criteria. The mean age of the participants was 51.5 ± 9.3 years. The presence of family history of hypertension (n=9) and personal history of alcohol consumption (n=3) and smoking (n=2) were registered; the rest of the participants did not show any of them. Baseline characteristics of women are described in Table 1; there were no significant differences between the placebo and the inulin groups. However, there was a significant difference in baseline glucose concentrations between the study groups. Despite this, SBP in the inulin group was not affected by baseline blood glucose according to the Mantel-Haenszel test (*p*=0.646).

Table 2 shows the frequency of patients according to the classification of SBP, DBP, HR, BMI, and BFP, as well as the histological type of breast cancer. However, these variables were not significantly different between the two types of breast cancer. The number of women with elevated SBP (>140 mmHg) was higher in those with ductal carcinoma (n=32) than those with lobular carcinoma (4). However, there was no significant difference between the SBP categories and the type of breast cancer* (p*=0.123). There were 3 women with ductal carcinoma and DBP >90 mmHg, but there were no women with lobular carcinoma and this level of DBP. Women with ductal carcinoma (n=10) presented a higher frequency of elevated MAP (>102.2) than those with lobular carcinoma (n=3). Nonetheless, only one patient with ductal carcinoma presented with an elevated HR (>100.1 BPM). In addition, patients were classified according to their BMI; they were grouped into the following categories: underweight (n=2), overweight (n=15), and obesity (n=11) in both study groups. Most of the participants showed a high BFP (n=32,* p*=0.180). The frequency was higher in the ductal carcinoma group.

Moreover, the frequencies of women under placebo and inulin supplementation with high or low blood pressure, HR, BMI, and BFP are shown in Table 3. These were not significantly different between groups. There were 6 women with SBP >130 mmHg in the placebo group and 13 in the inulin group before supplementation. After 21 days of supplementation, in the inulin group the number of participants with SBP >130.1 mmHg decreased from 13 to 10, but in the placebo group the number increased from 6 to 8. The number of patients with DBP >90 mmHg increased (n=4) in the placebo group; meanwhile in the inulin group, most of them remained under 90 mmHg (n=18). In the inulin group, all participants reached a MAP<102 mmHg (n=19) and HR <100 bpm after supplementation. Regarding BMI, the frequencies were not significantly different among the listed categories. Moreover, in both groups the number of women with high BFP was nonsignificantly changed; however, in the inulin group the number of patients with high BFP decreased.

In Table 4, Student's t analysis revealed that in the placebo group, SBP (+5.42 mmHg,* p*<0.001), DBP (+1.96 mmHg,* p*=0.003), MAP (+4.48 mmHg,* p*<0.001), and HR (+5.32 bpm,* p*<0.001) increased significantly, but BMI (-1.15 kg/m^2^,* p*<0.001) and BFP decreased (-1.99%,* p*<0.001) after supplementation. Nonetheless, in the inulin group, SBP (-4.21 mmHg,* p*<0.001) and HR (-0.12 bpm,* p*<0.001) decreased significantly. DBP in the inulin supplemented group increased as that in the placebo group did (+1.95 mmHg,* p*=0.008), but the increase in MAP (+1.11 mmHg,* p*=0.003) was not as high as the placebo group. Cohen's* d* values were lower in the inulin group than the placebo group for SBP (-0.27), DBP (0.18), MAP (0.11), and HR (-0.01). Effect size was determined by Cohen's* d* test and it revealed that inulin supplementation generated a small effect size (*d*<0.5) in SBP, DBP, MAP, HR, BMI, BFP, LBM, and SMM; nonetheless, supplementation in the placebo group exerted a medium size effect (*d*=0.75). Furthermore, inulin supplementation produced a significant increase in BMI (+1 kg/m^2^,* p*<0.001) with a nonsignificant loss of BFP (-3.23 %,* p*=0.288). There was also an increase in LBM (+2.61,* p*=0.427) and a minimum change in SMM (+0.16,* p*=0.723) after 21 days of inulin supplementation, but they were not statistically significant.

In addition, the Mantel-Haenszel test was applied to assess whether confounding variables could influence inulin supplementation net effect. The analysis showed that any of the following variables had a significant influence on the reduction of blood pressure by inulin supplementation: age (*p*=0.336), BMI (*p*=0.910), BFP (*p*=0.054), menopause status (*p*=0.280), tobacco use (*p*=0.456), general use of chronic medication (*p*=0.520), use of antihypertensive medications (*p*=0.874), vomiting presence (*p*=0.511), and constipation (*p*=0.483).

Regarding dietary adequacy, energy requirement and energy intake in 24 hours were calculated according to reported food equivalents, indicating that 44.8% (n=13) of the patients had a normal energy intake, 24.1% (n=7) had a low intake, and 31.0% (n=9) had a high intake; however, this is not a direct indicator of quality of the diet. Moreover, the presence of nausea and vomiting in the placebo group was 13.2% (n=5) at baseline and 23.7% (n=9) after the supplementation period. In the inulin group, the frequencies were 10.5% (n=4) and 13.2 % (n=5), respectively. Finally, the presence of constipation in the placebo group was 21.1% (n=8) at baseline and 23.7% (n=9) after supplementation and in the inulin group 26.3% (n=10) and 10.5% (n=4), respectively. Despite the fact that the presence of these events was lower in the inulin group, it was not significantly different from the placebo group.

## 4. Discussion

In this double-blind placebo controlled trial, we found that supplementation with inulin reduces SBP and attenuates increases in DBP and MAP in women with breast cancer under neoadjuvant therapy. Similar to our findings, some trials have shown that supplementation with dietary fiber promotes the reduction of blood pressure although there are some differences associated with the time of supplementation and the change in blood pressure. For instance, fiber intake for 8 weeks nonsignificantly reduces SBP (-1.15 mmHg) and significantly reduces DBP (-1.65 mmHg) [29]. In contrast, in our study we found that the group supplemented with inulin for 21 days has a significant reduction in SBP (-4.21 mmHg), but not in DBP. Brazilian patients with type 1 diabetes mellitus, who consume the recommended fiber amount (≥14g/1000 kcal), present lower SBP and DBP than patients who consume less than that [30], and this is not influenced by sex. These differences in blood pressure could be due to the physiological mechanisms induced specifically by SCFA derived from inulin metabolism in the gut. One limitation of our study is that diet components associated with modifications in blood pressure like saturated fats or sodium as well as fiber intake were not completely monitored during the supplementation. Despite that limitation, patients had general nutritional counselling by the hospital nutritional staff throughout the treatment. In addition, in some trials with inulin-type fructans, there are no differences in appetite and energy intake in adult women or men [31].

One of the main findings in this study is that the effect of inulin supplementation on SBP is more pronounced than the one on DBP. This is consistent with experimental studies where SBP, systolic volume, and left ventricular diameter in systole were lower in rats fed with a high-fat diet and supplemented with inulin than in unsupplemented rats fed only with the high-fat diet [32]. Therefore, it is plausible that this type of cardiovascular responses could be due to changes in sympathetic activity and renin-angiotensin system mediated by SCFA, which are derived from inulin metabolism in gut microbiota. Once SCFA are absorbed and reach the circulation, they bind to G-protein-coupled receptors (GPCRs) in vascular smooth muscle cells and renal arterioles modulating signaling pathways that control blood pressure [33–35]. In fact, the group supplemented with inulin showed a diminished HR which might be a response modulated by this type of GPCRs and SCFA [36]. Therefore, the use of prebiotics and SCFA could be a potential aid in the treatment and evolution of patients with intestinal microbiota imbalance, which is being identified as a causal factor of hypertension and a consequence of chemotherapy [37, 38].

Supplementation with 10 and 20 g of inulin for 3 weeks has been linked to decreases in triacylglycerols and hypercholesterolemia in men [15, 39]. In our study, blood triacylglycerols or cholesterol were not measured, and this restricts the conclusion about inulin influence on dyslipidemia, atherosclerosis, and prevention of CVD risk. However, there is evidence that inulin supplementation does not change cholesterol and triacylglycerols in women without cancer [40] and with breast cancer during chemotherapy with cyclophosphamide and doxorubicin; instead, HDL-c and apolipoprotein A1 could be reduced [41]. Furthermore, inulin supplementation for 12 weeks decreases DBP and SBP without modifications in serum cholesterol, triacylglycerols, and high-density lipoprotein cholesterol in hypertensive men and women [42]. Future studies would be needed to explain how hypertension and breast cancer could be associated and confirm whether cardiovascular benefits of inulin remain or are improved over a longer period of supplementation.

It is important to mention that there is any statistical influence from other confounding variables such as changes in BMI, glucose concentrations, age, menopause status, vomiting, constipation, and use of antihypertensive drugs. This suggests that the decrease in SBP in the group supplemented with inulin is due to the supplementation itself. Furthermore, creatinine clearance (Table 1) and hepatic function were under the expected level to carry out neoadjuvant therapy cycles. These findings agree with previous evidence that most antihypertensive drugs are not associated with breast cancer risk, except for the use of calcium channel blockers that might increase the risk of ductal and lobular carcinoma [43]. In Table 2, the frequency of high SBP, BMI, and BFP is higher in patients diagnosed with ductal carcinoma. However, the number of participants with this type of cancer is also higher than the group with lobular carcinoma; therefore, a clear association between the histological type of cancer and inulin effect on blood pressure may not be suitable to establish at this point.

Another interesting finding is the contrasting effect of inulin on BMI and BFP. At the beginning of our study, 68.4% of the patients were overweight and obese, 89.4% had a high BFP, and 34.2% had a MAP above 120 mmHg. However, the frequency of women with overweight or obesity is nonsignificantly different after 21 days of supplementation between both groups (Table 3). Also, BMI increases after inulin supplementation, but BFP is nonsignificantly reduced (Table 4). In contrast to this, BMI is reduced after inulin supplementation in women with type 2 diabetes mellitus and obesity [23, 44], but in these studies the supplementation was conducted for a longer period of time. Furthermore, BFP was reduced after the first cycle of chemotherapy in the placebo group although it was nonsignificantly reduced in the inulin group. This is consistent with the Mantel-Haenszel analysis, regarding the influence of BFP on the reduction of blood pressure because the* p* value was greater than 0.05 in the group supplemented with inulin, but it was lower in the placebo group. This also suggests that BMI and BFP may not exert an influence on blood pressure after inulin supplementation. Increases in BFP are usually associated with hypertension and breast cancer metastasis [45–47], but in this study we found that BMI and BFP are reduced after the first cycle of chemotherapy in the placebo group. In contrast, in the inulin group, BMI increases and BFP is diminished. The increase in BMI could be because of the increase in LBM but not in SMM in the group supplemented with inulin (Table 4). Regarding this, a longitudinal study in African American women showed that central adiposity, reflected in waist circumference, has a stronger association with hypertension than BFP which is a total adiposity parameter [48]. In our study, there is no significant difference in the waist-hip ratio obtained by electric bioimpedance (data not shown). In consequence, the effect of inulin on central adiposity cannot be completely explained in this study, and it might not have a direct association with modifications in blood pressure.

## 5. Conclusion

Hypertension and breast cancer are multifactorial diseases that involve complex molecular and physiological mechanisms. Besides that, there is little evidence about the effects of prebiotics on these two diseases and particularly in women with breast cancer under neoadjuvant therapy. This is the first double-blind placebo controlled clinical trial in women where the supplementation with inulin elicits a reduction in SBP and HR and prevents increases in DBP. Furthermore, inulin supplementation nonsignificantly reduces BFP and increases BMI. Therefore, this might be a novel nutraceutical approach to improve the quality of life and cardiovascular health of women who are at early stages of breast cancer and who are under this type of therapy. Consequently, supplementation with inulin might help to improve the prognosis of women with breast cancer and CVD risk. For future experimental or clinical trials, it would be interesting to consider the assessment of variables (food patterns, physical activity, intestinal microbiota, and immune and biochemical markers) that could further help to explain the physiological mechanisms by which inulin elicits beneficial responses as well as to increase the supplementation period and sample size.

## Figures and Tables

**Table 1:** Baseline characteristics of women by study group.

	Group		
Measurements	Placebo (n=19)	Inulin (n=19)	*p*
Age (years)	51.89 (10.36)	51.58 (8.45)	0.919
Weight (kg)	61.97 (10.90)	69.16 (13.77)	0.083
Fasting glucose			
Normal	11 (28.9)	4 (10.5)	0.045
High	8 (21.1)	15 (39.5)	
Creatinine clearance			
Low	8 (21.1)	7 (18.4)	0.999
Normal	11 (28.9)	12 (31.6)	
Menopause			
Premenopause	9 (23.7)	7 (18.4)	0.743
Postmenopause	10 (26.3)	12 (31.6)	
Chronic medication			
Yes	7 (18.4)	12 (31.6)	0.194
No	12 (31.6)	7 (18.4)	
HTA medication			
Yes	4 (10.5)	10 (26.3)	0.091
No	15 (39.5)	9 (23.7)	

Data: media (standard deviation); Student's t-test: *∗p*<0.05.

n (%), *χ*^2^ test: *∗p*<0.05.

Fasting glucose: normal ≤99 mg/dl; high ≥100 mg/dl.

Creatinine clearance: normal ≥90 ml/min; low ≤89 ml/min.

HTA: hypertension.

**Table 2:** Frequency of patients according to baseline classification of blood pressure, BMI, and BFP vs. the type of breast cancer.

Breast Cancer Diagnosis			
Measurements	Ductal	Lobular	*p*
	(n=32)	(n=6)	
SBP (mmHg)			
≤120	5	2	0.123
120.1-130	12	0	
130.1-140	5	0	
≥140.1	10	4	

DBP (mmHg)			
<89	29	6	0.435
>90	3	0	

MAP (mmHg)			
<102	22	3	0.374
>102.1	10	3	

HR (BPM)			
<100	31	6	0.661
>100.1	1	0	

BMI (kg/m^2^)			
Underweight	1	1	0.779
Normal	8	2	
Overweight	13	2	
Obese Class I	8	1	
Obese Class II	1	0	
Obese Class III	1	0	

BFP (%)			
Normal	5	1	0.999
High	27	5	

SBP: systolic blood pressure; DBP: diastolic blood pressure; MAP: mean arterial pressure; HR: heart rate; BMI: body mass index; BFP: body fat percentage. BFP: normal ≤29.9; high ≥30.

*χ* ^2^ test: *∗p*<0.05.

**Table 3:** Frequency of women according to classification of blood pressure, HR, BMI, and BFP vs. supplementation group.

	Placebo (n=19)		Inulin (n=19)		*p*	
	Baseline	After	Baseline	After	Baseline	After
SBP (mmHg)						
≤ 120	5	6	2	2	0.071	0.251
120.1-130	8	5	4	7		
130.1-140	0	2	5	5		
≥140.1	6	6	8	5		

DBP (mmHg)						
< 89	17	15	18	18	0.999	0.340
> 90	2	4	1	1		

MAP (mmHg)						
< 102	12	17	13	19	0.999	0.486
>102.1	7	2	6	0		

HR (BPM)						
< 100	19	18	18	19	0.999	0.486
> 100.1	0	1	1	0		

BMI (kg/m^2^)						
Underweight	1	2	1	0	0.628	0.418
Normal	6	6	4	5		
Overweight	8	9	7	7		
Obese Class I	3	2	6	4		
Obese Class II	1	0	0	1		
Obese Class III	0	0	1	1		

BFP (%)						
Normal	5	5	1	4	0.180	0.999
High	14	14	18	15		

SBP: systolic blood pressure; DBP: diastolic blood pressure; MAP: mean arterial pressure; HR: heart rate; BMI: body mass index; BFP: body fat percentage. BFP: normal ≤29.9; high ≥30.

*χ* ^2^ test: *∗p*<0.05.

**Table 4:** Effect of inulin supplementation on blood pressure, heart rate, BMI, and BFP.

	Groups											
	Placebo						Inulin					
	(n=19)						(n=19)					
	Baseline		Before				Baseline		Before			
Measurements	Mean	SD	Mean	SD	Effect Size	*p* value	Mean	SD	Mean	SD	Effect Size	*p* value
SBP (mmHg)	130	19.41	135.42	22.55	0.28	<0.001	140.21	15.66	136	14.09	-0.27	<0.001
DBP (mmHg)	80.36	7.36	82.32	7.00	0.27	0.003	75.21	10.65	77.16	7.47	0.18	0.008
MAP	96.91	10.12	101.39	12.21	0.44	<0.001	79.84	10.23	80.95	9.36	0.11	0.003
HR (bpm)	75.15	7.13	80.47	10.40	0.75	<0.001	96.87	11.45	96.75	8.46	-0.01	<0.001
BMI (kg/m^2^)	26.19	5.54	25.04	4.68	-0.21	<0.001	27.7	8.45	28.7	5.78	0.12	<0.001
BFP (%)	38.96	11.55	36.97	11.68	-0.17	0.001	41.74	7.63	38.51	8.18	-0.42	0.288
LBM (kg)	38.38	4.08	38.85	4.28	0.12	0.440	40.21	11.46	42.82	4.99	0.23	0.427
SMM (kg)	20.88	2.4	21.81	4.56	0.39	0.411	22.65	3.31	22.49	3.51	-0.05	0.723

SBP: systolic blood pressure; DBP: diastolic blood pressure; MAP: mean arterial pressure; HR: heart rate; BMI: body mass index; BFP: body fat percentage; LBM: lean body mass; SMM: skeletal muscle mass. Student's t-test: *∗p*<0.05.

## Data Availability

The data used to support the findings of this study are included within the article.
